# Immune cell composition of the bronchoalveolar lavage fluid in healthy and respiratory diseased dromedary camels

**DOI:** 10.1186/s12917-022-03446-7

**Published:** 2022-09-21

**Authors:** Turke Shawaf, Hans-Joachim Schuberth, Jamal Hussen

**Affiliations:** 1 Department of Clinical Sciences, College of Veterinary Medicine, King Faisal University, Al-Ahsa, Saudi Arabia; 2 Institute for Immunology, University of Veterinary Medicine Hannover, Foundation, Hannover, Germany; 3 Department of Microbiology, College of Veterinary Medicine, King Faisal University, Al-Ahsa, Saudi Arabia

**Keywords:** Bronchoalveolar lavage, Leukocytes, Camel, Flow cytometry, Macrophages, Mucosal immunology

## Abstract

**Background:**

Respiratory diseases are among the most common and expensive to treat diseases in camels with a great economic impact on camel health, welfare, and production. Bronchoalveolar lavage fluid (BALF) has been proven as a valuable sample for investigating the leukocyte populations in the respiratory tract of several species. In the present study, fluorescent antibody labeling and flow cytometry were used to study the immune cell composition of BALF in dromedary camels. Animals with clinical respiratory diseases (*n* = seven) were compared with apparently healthy animals (*n* = 10). In addition, blood leukocytes from the same animals were stained in parallel with the same antibodies and analyzed by flow cytometry.

**Results:**

Camel BALF macrophages, granulocytes, monocytes, and lymphocytes were identified based on their forward and side scatter properties. The expression pattern of the cell markers CD172a, CD14, CD163, and MHCII molecules on BALF cells indicates a similar phenotype for camel, bovine, and porcine BALF myeloid cells. The comparison between camels with respiratory disease and healthy camels regarding cellular composition in their BALF revealed a higher total cell count, a higher fraction of granulocytes, and a lower fraction of macrophages in diseased than healthy camels. Within the lymphocyte population, the percentages of helper T cells and B cells were also higher in diseased than healthy camels. The elevated expression of the activation marker CD11a on helper T cells of diseased camels is an indication of the expansion of helper T cells population due to infection and exposure to respiratory pathogens. The higher abundance of MHCII molecules on BALF macrophages from diseased camels indicates a polarization toward an inflammatory macrophage phenotype (M1) in respiratory diseased camels. No significant differences were observed in the systemic leukogram between healthy and diseased animals.

**Conclusions:**

Collectively, the current study represents the first report on flow cytometric analysis of immune cell composition of bronchoalveolar lavage fluid (BALF) in dromedary camels.

## Background

Respiratory diseases are among the most common and expensive to treat diseases in camels with a high economic impact on camel health, welfare, and production [1–7]. Causative agents of camel respiratory infectious diseases mainly include viral and bacterial pathogens [8]. Parainfluenza 3 virus [9], bovine respiratory syncytial virus, Pasteurella spp., and Corynebacterium spp. [10, 11] are among the main pathogens isolated from camels with respiratory disease. Camels can be infected by the Middle East respiratory syndrome coronavirus (MERS-CoV), which does not lead to the development of clinical disease in camels; however, it can be transferred to humans from infected dromedary camels [12].

Bronchoalveolar lavage (BAL) is a useful procedure to explore large areas of the respiratory tract [13–17]. Cytological, microbiological, and immunological evaluation of BALF enables the detection of subclinical respiratory disease and the identification of the severity grade, and stage of inflammatory reactions in the respiratory tract [18, 19]. BALF has been proven as a valuable sample for investigating tissue resident immune cells in the respiratory tract of several species including mankind [17], horses [20], cattle [21, 22], sheep [23], alpaca [24], pigs [25], dogs [26, 27], and cats [28]. Also in the dromedary camel, the BAL procedure has been recently used for the collection of samples for cytological analysis [29].

Alveolar macrophages, lymphocytes, and neutrophils are major immune cell populations of the BALF [30, 31]. The dominant cell type is alveolar macrophages, which represent long-living effector cells residing within the alveoli and can clear pathogens rapidly using their different and elastic antimicrobial functions [32–34]. During the late phase of infection, alveolar macrophages contribute to the resolution of inflammation and restoring homeostasis by clearing cell debris and apoptotic neutrophils [33, 35]. The current functional classification of macrophages distinguishes two main subsets, the M1 classically-activated macrophages, and the M2 alternatively-activated macrophages. While the polarization towards M1 macrophages is guided by pro-inflammatory stimuli like bacterial lipopolysaccharide (LPS) and the T helper (Th) 1 cytokine interferon γ (IFN-γ) [36], M2 macrophage polarization requires type 2 cytokines like the Th2 cytokines IL-4 and IL-13 [37]. Classically-activated M1 macrophages have the potential to kill intracellular pathogens and contribute to the early inflammatory response through the production of pro-inflammatory cytokines such as tumor necrosis factor alpha (TNF-α) and interleukin 12 (IL-12). On the other side, M2 macrophages are anti-inflammatory cells that play a role in the resolution of inflammation and wound healing by producing the immunoregulatory cytokine IL-10 [37]. Macrophage subsets can be distinguished according to their specific surface markers. Major histocompatibility complex (MHC) class-II molecules have been identified as a marker of classical macrophages, whereas cluster of differentiation (CD)163 molecules are a characteristic marker for M2 macrophages with anti-inflammatory properties [38]. Both macrophage subsets could be identified in the human BALF [35].

Marker surface antigens expressed by myeloid immune cells in the BALF include CD172a, CD14, CD163, and MHC class II molecules. The CD172a protein, which is also known as the signal regulatory protein α (SIRP α), is a molecule with inhibitory function expressed on all cells of the myeloid lineage including macrophages, neutrophils, and monocytes [39, 40]. The molecule CD14 is a well-known receptor for the recognition of the cell-wall component of gram-negative bacteria, LPS, and is mainly expressed on macrophages and monocytes [41]. The CD163 antigen, which is exclusively expressed by macrophages and monocytes, is the receptor for binding and uptake of hemoglobin-haptoglobin complexes [42]. The MHC II molecules are antigen receptors involved in the presentation of exogenous peptide antigens to the T cell receptor and the subsequent activation of antigen-specific T helper cells [43, 44].

Flow cytometry is a methodology that has proven highly successful in characterizing cells in different organ systems. It provides the possibility to identify, quantify, phenotype, and isolate individual cell subsets. Using this technique, different staining panels for immunophenotyping of leukocytes have enabled the rapid and detailed characterization of immune responses to vaccination or infection [45, 46]. For humans, cattle, pigs, and horses, the tissue-resident immune cells in the respiratory tract have been investigated by flow cytometric analysis of BALF [47–50], but little is known about the same in the dromedary camel. In the present study, fluorescent antibody labeling and flow cytometry were used to study the immune cell composition of BALF in dromedary camels. The percentages of several leukocyte populations were compared between animals with clinical respiratory diseases and apparently healthy animals.

## Methods

### Animals and clinical examination

The present study was conducted at the Veterinary Teaching Hospital of the King Faisal University in Al-Ahsa region (Al-Hofuf) in the Eastern Province of Saudi Arabia. Bronchoalveolar lavage fluid samples were collected from seventeen dromedary camels (*Camelus dromedaries*) of the Al-Majaheem breed including ten healthy camels (control group) and seven camels with clinical respiratory disease (diseased group). The camels of the control group (aged between eight and 11 years) were selected from the animals maintained at the Camel Research Center of the King Faisal University. The diseased group camels (aged between 10 and 14 years) were selected randomly from camels with clinical symptoms of respiratory disease, which were brought to the Veterinary Teaching Hospital, College of Veterinary Medicine, King Faisal University. All camels were tested for the zoonotic virus Middle East respiratory syndrome coronavirus (MERS-CoV) using the BIONOTE® Rapid MERS-CoV Ag Test Kit (BioNote Inc., Hwaseong, Gyeonggi, Republic of Korea) and nasal swabs [51] to exclude animals with MERS-CoV infection (zoonotic risk). Animal history and clinical examination signs were recorded for all animals. Camels with respiratory disease were identified based on abnormal respiratory signs such as cough, nasal discharge, dyspnea, or abnormal lung sounds [29].

### Blood collection and leukocytes separation

Blood samples were collected from all animals by puncture of the vena jugularis externa using vacutainer Ethylenediaminetetraacetic acid (EDTA) tubes (BD, Germany). Leukocytes were separated from blood samples after the removal of red blood cells by repeated cycles of hypotonic lysis. After dilution with phosphate-buffered saline (PBS) (1:9) in 15 ml falcon tubes, blood samples were centrifuged at 4 C° and 1000×g for 25 minutes. After plasma removal, the red blood cells were lysed by adding 5 mL distilled water for 20 s followed by the addition of the same volume of double concentrated PBS and centrifugation at 500×g and 4 C° for 10 min with a break. After re-suspending the cell pellet, the procedure was repeated to ensure the removal of all red blood cells (RBC). Subsequently, 10 mL PBS was added to the cells, and the cells were washed two times (250×g and 100×g for 10 min each). Finally, the cells were adjusted to 5 × 10^6^ cells/mL in cell staining buffer. The counting of total leukocytes in blood and BALF samples was estimated using Neubauer’s counting hemocytometer and microscopy. For blood samples, Türk Solution was added to lyse the RBC. For cell viability check, the DNA-binding dye propidium iodide (PI; 2 μg/mL, Calbiochem, Germany) was added to the separated cells followed by a flow cytometric analysis of PI uptake versus exclusion (FACSCalibur, Becton Dickinson Biosciences). The percentage of viable (PI-negative) cells was always above 93% of total blood leukocytes.

### Bronchoscopy and collection of bronchoalveolar lavage fluids (BALF)

Bronchoscopy and BALF collection were performed as previously described [29]. Camels were positioned in sternal recumbency position. After animal sedation by intravenous injection of 2% xylazine (Rompun, Bayer Health Care, Germany) at a dose of 0.1 mg per kg body weight, a 3.2 m long and 12 mm tip diameter bronchoscope (EVIS Olympus, Vienna, Austria) was introduced into the oral cavity. During the BAL procedure, 20–40 mL of 1% Lidocaine were infused into the lower airway to reduce coughing. A 240 cm long catheter (EQUIVET B.A.L., KRUUSE, Denmark) was introduced through the speculum of the mouth gag into the oral cavity until the pharynx and then advanced into the larynx, trachea, and bronchi until reaching a slight resistance. Pre-warmed (37 °C) sterile isotonic saline (250 ml) was instilled via the BAL catheter. The BALF was aspirated immediately after injection and the samples were positioned on ice and submitted to the lab within 30 min of collection. Flow cytometric analysis was conducted after 1 h of sample collection.

### Monoclonal antibodies

The antibodies used for cell staining are presented in Table 1.

**Table 1:** List of antibodies

Antigen	Antibody clone	Labeling	Source	Isotype
CD14	CAM36A	–	WSU	Mouse IgG1
CD14	Tuk4	PerCP	Thermofisher	Mouse IgG2a
MHCII	TH81A5	–	Kingfisher	Mouse IgG2a
CD172a	DH59b		WSU	Mouse IgG1
CD163	LND68A	–	Kingfisher	Mouse IgG1
CD4	GC50A1	–	WSU	Mouse IgM
WC1	BAQ128A	–	WSU	Mouse IgG1
CD11a	HUH73A	–	WSU	Mouse IgG1
B cell antigen	GC26A	–	WSU	Mouse IgM
Mouse IgM	poly	APC	Invitrogen	Goat IgG
Mouse IgG1	poly	FITC	Invitrogen	Goat IgG
Mouse IgG2a	poly	PE	Invitrogen	Goat IgG

*WSU* Washington State University, *PerCP* Peridinin-Chlorophyll-Protein, *MHC* Major Histocompatibility Complex, *WC1* Workshop cluster 1, *APC* Allophycocyanin, *FITC* Fluorescein isothiocyanate, *PE* Phycoerythrin, *poly* Polyclonal

All monoclonal antibodies were directed against leukocyte antigens of other animals including bovine (CD14, CD163, CD4, WC1, CD11a), and swine (MH II). All antibodies were tested for reactivity against camel leukocytes in previous studies [52–56].

### Cell labeling and flow cytometry

Cell labeling and flow cytometric analysis of BALF and blood samples were performed as previously described [57]. Separated BALF or blood leukocytes were incubated for 20 min at 4 °C with unlabeled antibodies (Table 1) to the cell marker antigens CD172a, CD14, CD163, MHC-class II, CD4, WC1, GC26A or with directly labeled antibodies to CD14 or the cell adhesion molecule CD11a. After two washing steps (by adding 150 μl washing buffer followed by centrifugation at 300 xg for 3 min), mouse secondary antibodies (IgG1, IgG2a, and IgM; Invitrogen) labeled with different fluorochromes were added to the cells followed by incubation for 20 min at 4 °C. Staining with mouse isotype control antibodies (BD, Biosciences) was also performed. After the final cell wash, labeled cells were analyzed by flow cytometry (FACSCalibur, Becton Dickinson Biosciences) by the acquisition of at least 100,000 total blood leukocytes or 10,000 BALF cells. Collected data were analyzed with the FlowJo software (FLOWJO, LLC).

### Molecular detection of selected respiratory viruses in BAL fluid

Collected BALF samples were tested for the bovine parainfluenza 3 virus and bovine respiratory syncytial virus by reverse transcription-polymerase chain reaction (RT-PCR) using primers shown in Table 2.

**Table 2:** List of primers

Target	Sequence	Expected product (bp)	Annealing Temp.
BRSV	F: 5ʹ-CAT CAA TCC AAA GCA CCA CAC TGT C-3ʹ	381 bp	62 °C
	R: 5ʹ-GCT AGT TCT GTG GTG GAT TGT TGT C -3ʹ		
BPIV-3	F: 5ʹ-AGT GAT CTA GAT GATGAT CCA-3ʹ	328 bp	47 °C
	R: 5ʹ-GTT ATT GAT CCA ATT GCT GT-3ʹ		

*BRSV* Bovine respiratory syncytial virus, *BPIV-3* Bovine parainfluenza virus type 3, *F* Forward, *R* Revers [58, 59]

Total RNA extracted from BALF utilizing QIAamp Viral RNA Mini Kit (QIAGEN, USA) according to manufactures instructions. The extracted RNAs were subjected to RT-PCR using One-step RT-PCR Kit (QIAGEN, USA). Briefly, the amplification reaction was performed in a 25 μl RT-PCR reaction mixture including 5 μl of the total RNA, 5 μl of the 5x Qiagen one-step RT-PCR buffer, 5 μl of the Q buffer, 1 μl of a dNTPs mix, 1 μl (50 pmol) of each primer, 1 μl of the enzyme mix, and 6 μl of RNase free water. The RT-PCR reaction was performed at 50 °C for 30 min, then 95 °C for 15 min, followed by 40 cycles consisting of denaturing step at 95 °C for 30 seconds, primers annealing temperature according to Table 2 for 30s and 72 °C for 30 seconds and final extension step at 72 °C for 10 min. The amplified PCR was electrophoresed in 1.2% agarose gel containing 0.5 μg/ml ethidium bromide and analyzed using an ultraviolet gel documentation system (BIORAD) [58, 59].

## Statistical analyses

Statistical analysis was performed using the statistical software program Prism (GraphPad software version five, GraphPad Software, San Diego, CA, USA). The results were presented as means ± standard error of the mean (SEM). Data normal distribution was evaluated using the Kolmogorov–Smirnov test (with the Dallal–Wilkinson–Lilliefor *p*-value). The unpaired student’s t-test or the Wilcoxon test were used to comparing the two groups for normally distributed data or for data that failed to pass the normality test, respectively. *P*-values < 0.05 were considered significant.

## Results

### Main leukocyte populations in the bronchoalveolar lavage fluid (BALF) from healthy and respiratory diseased camels

**Fig. 1:**
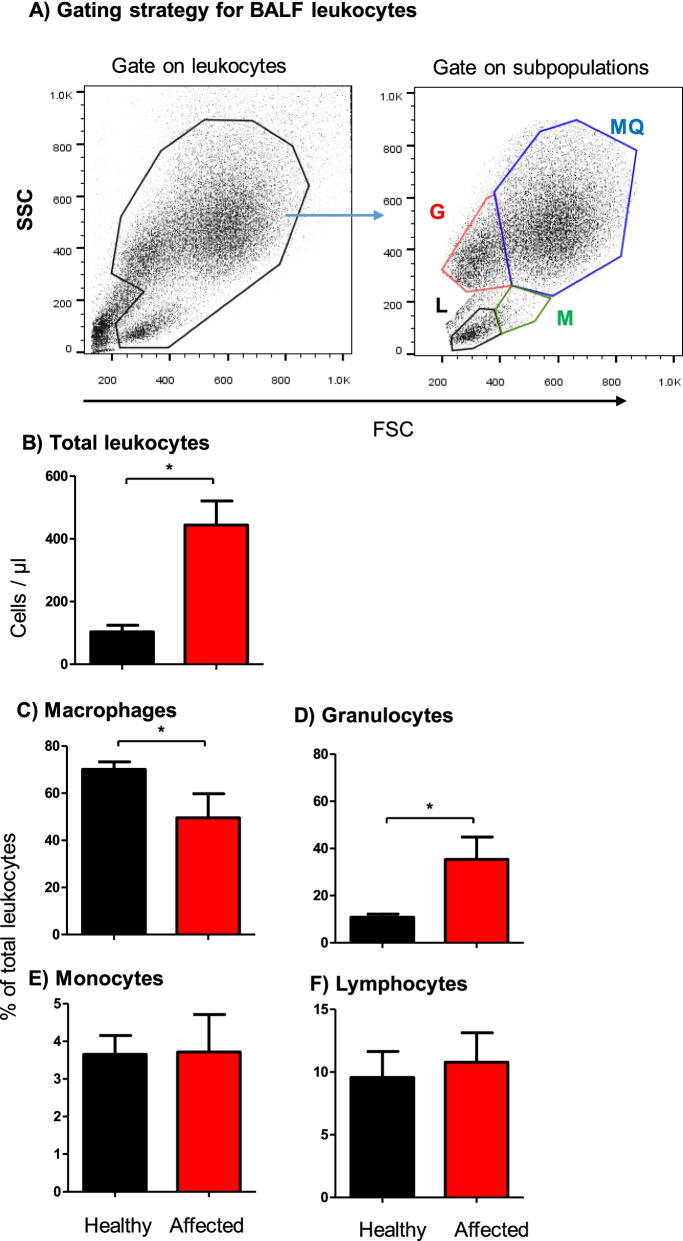
Flow cytometric analysis of leukocyte populations in the bronchoalveolar lavage fluid (BALF) of healthy and diseased camels. **A** Gating strategy for the identification of BALF leukocytes. Camel BALF leukocytes were classified based on their forward (FSC) and side (SSC) light scattering characteristics into a major population of FSC^high^/SSC^high^ macrophages, a smaller population of FSC^low^ SSC^high^ granulocytes, and two minor populations of FSC^high^ SSC^low^ monocytes and FSC^low^ SSC^low^ lymphocytes. **B** Total leukocyte count and the percentage of macrophages (**C**), granulocytes (**D**), monocytes (**E**), and lymphocytes (**F**) in BALF from healthy and diseased camels were calculated and presented graphically as mean ± SEM. * indicates a significant difference between the two groups (*p* < 0.05; t-test)

According to a previously described gating strategy for leukocytes in the bovine bronchoalveolar lavage fluid (BALF) [48], camel BALF leukocytes were classified based on their forward (FSC) and side (SSC) light scattering characteristics into four main populations: A major population (mean ± SEM = 70.1 ± 3.2% of total BALF leukocytes) of FSC^high^ SSC^high^ macrophages, a smaller population of FSC^low^ SSC^high^ granulocytes (12.7 ± 1.7% of total BALF leukocytes), and two minor populations of FSC^high^ SSC^low^ monocytes (3.6 ± 0.5% of total BALF leukocytes) and FSC^low^ SSC^low^ lymphocytes (9.6 ± 2.0% of total BALF leukocytes) (Fig. 1A). BALF samples from respiratory diseased camels contained significantly (*p* = 0.0007) higher numbers of total leukocytes (444.3 ± 77.2 cell/μl) than clinically healthy camels (103.5 ± 21.3 cell/μl) (Fig. 1B). Within the BALF leukocyte population of diseased camels, there was an expansion in the fraction of granulocytes (35.4 ± 9.4% compared to 12.7 ± 1.7% of total BALF leukocytes in healthy camels) with a reduced fraction of macrophages (49.6 ± 10.2% compared to 70.1 ± 3.2% of total BALF leukocytes in healthy camels) compared to healthy animals. The percentages of lymphocytes and monocytes did not differ significantly (*p* > 0.05) between the two groups (Fig. 1C-F).

### Blood leukocyte composition in healthy and respiratory diseased camels

**Fig. 2:**
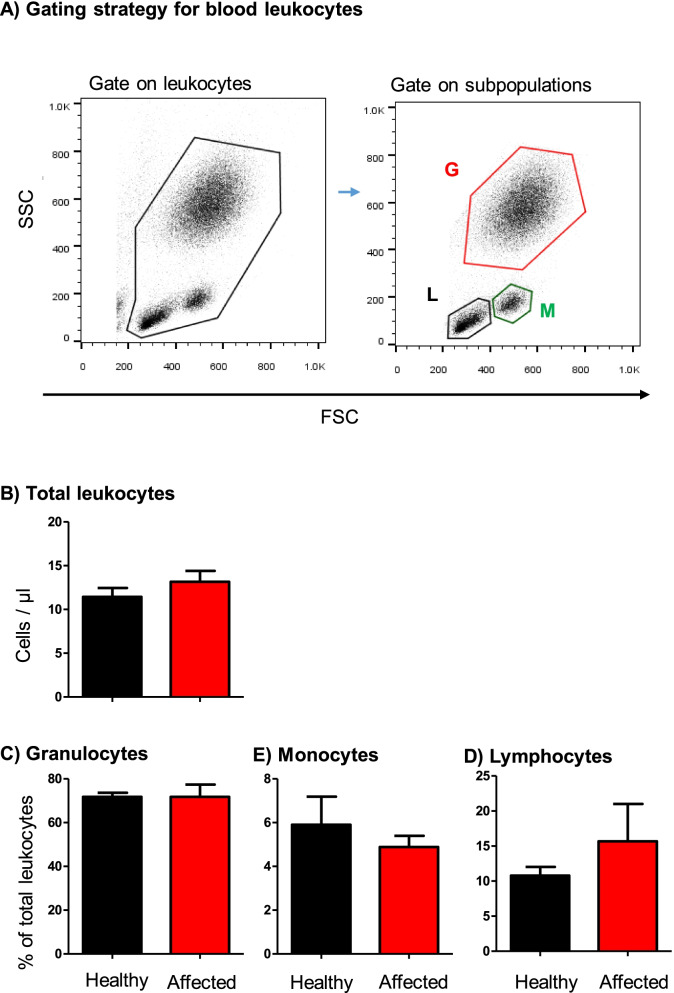
Flow cytometric analysis of leukocyte populations in the blood of healthy and diseased camels. **A** Gating strategy for the identification of blood leukocytes. Camel blood leukocytes were classified based on their forward (FSC) and side (SSC) light scattering characteristics into FSC^low^/SSC^high^ granulocytes, FSC^high^ SSC^low^ monocytes, and FSC^low^ SSC^low^ lymphocytes. **B** Total leukocyte count and the percentage of granulocytes, monocytes, and lymphocytes in blood from healthy and diseased camels were calculated and presented graphically as mean ± SEM. * = *p* < 0.05

With a mean ± SEM of 13.2 ± 1.2 cell/μl blood, the total leukocyte count in blood samples collected from camels with the respiratory disease did not differ significantly (*p* > 0.05) from the total leukocyte count in blood from healthy camels (11.5 ± 1.1). This was also the case for the differential leukocyte composition in blood with comparable (*p* > 0.05) fractions of granulocytes, lymphocytes, and monocytes in the two animal groups (Fig. 2). In addition, there was no correlation between the leukocyte count in blood and BALF, neither for each animal group separately nor for the two groups together (r square = 0.12).

### Lymphocyte composition and phenotype in BALF from healthy and diseased camels

**Fig. 3:**
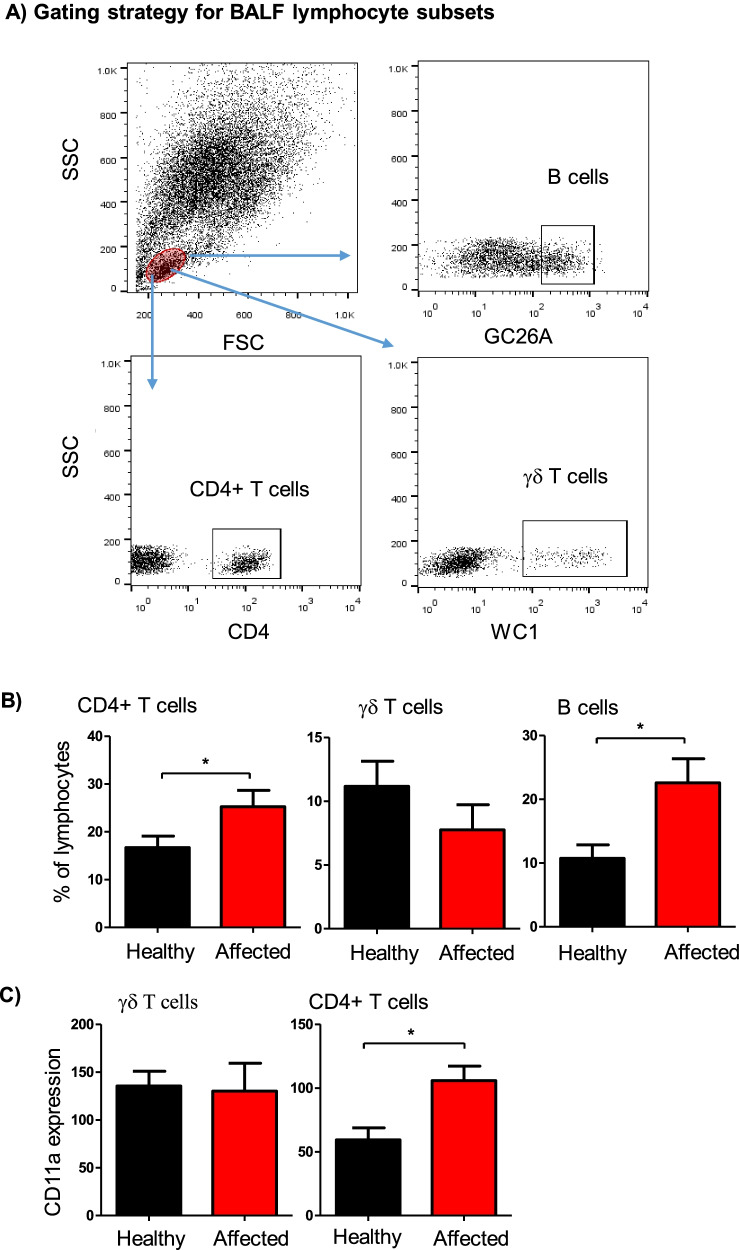
Flow cytometric analysis of selected lymphocyte subsets in BALF from healthy and diseased camels. **A** Gating strategy for the identification of BALF lymphocyte subsets. Camel BALF leukocytes were stained with antibodies to CD4, the B cell marker GC26A, WC1, and LFA-1 (CD11a), and stained cells were analyzed by flow cytometry. **B** The percentage of helper T cells, B cells, and γδ T cells within total BALF lymphocytes were calculated for healthy and diseased camels and presented graphically. **C** The figure presents the expression level of CD11a on CD4+ helper T cells and γδ T cells. * = *p* < 0.05

Flow cytometric analysis of selected lymphocyte subsets in BALF samples identified significant differences (*P* < 0.05) between healthy and diseased camels (Fig. 3A). BALF samples from camels with clinical respiratory disease contained higher percentages of CD4+ T helper cells (25.2 ± 3.4% of total lymphocytes versus 16.7 ± 2.3% in healthy animals; *p* = 0.02) and B cells (22.6 ± 3.8% of total lymphocytes versus 10.7 ± 2.1% in healthy animals; *p* = 0.006) when compared to lymphocyte composition in healthy animals (Fig. 3B). In addition, helper T cells in BALF samples from diseased animals expressed higher levels of the cell adhesion molecule lymphocyte function-associated antigen 1 (LFA-1; CD11a) than healthy camels (Fig. 3C). The percentage of WC1-positive gamma delta (γδ) T cells did not differ significantly between the two groups.

### Lymphocyte composition and phenotype in blood from healthy camels and camels with respiratory disease

**Fig. 4:**
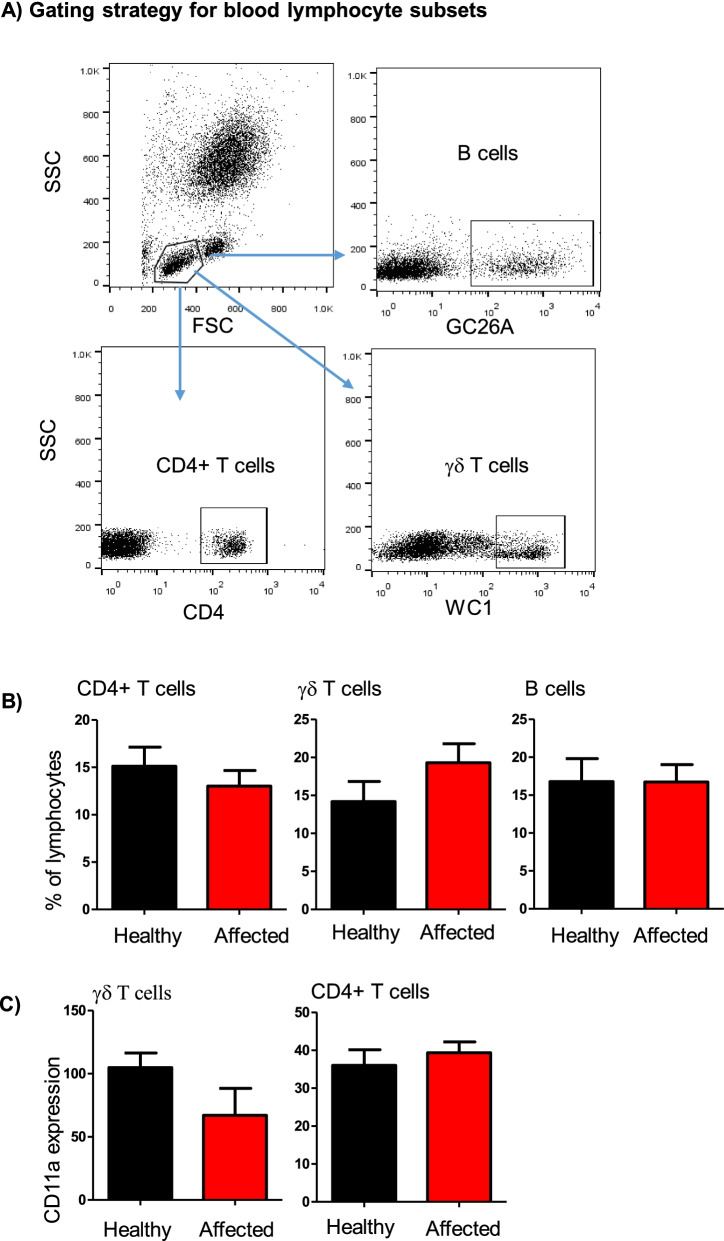
Flow cytometric analysis of selected lymphocyte subsets in blood from healthy and diseased camels. **A** Gating strategy for the identification of blood lymphocyte subsets. Camel blood leukocytes were stained with antibodies to CD4, the B cell marker GC26A, WC1, and CD11a (LFA-1). Stained cells were analyzed by flow cytometry. **B** The percentage of helper T cells, B cells, and γδ T cells within total blood lymphocytes were calculated for healthy and diseased camels and presented graphically C) the expression level of CD11a on helper T cells and γδ T cells was calculated as mean fluorescence intensity and presented as mean ± SEM. * = *p* < 0.05

Within blood lymphocytes, the fraction of helper T cells, B cells, and γδ T cells did not differ significantly (*p* > 0.05) between healthy and affected camels (Fig. 4A-B). Similarly, the abundance of LFA-1 on CD4+ helper T cells and γδ T cells was comparable in healthy and diseased animals (Fig. 4C).

### The immunophenotype of BALF myeloid cells from healthy camels and camels with respiratory disease

**Fig. 5:**
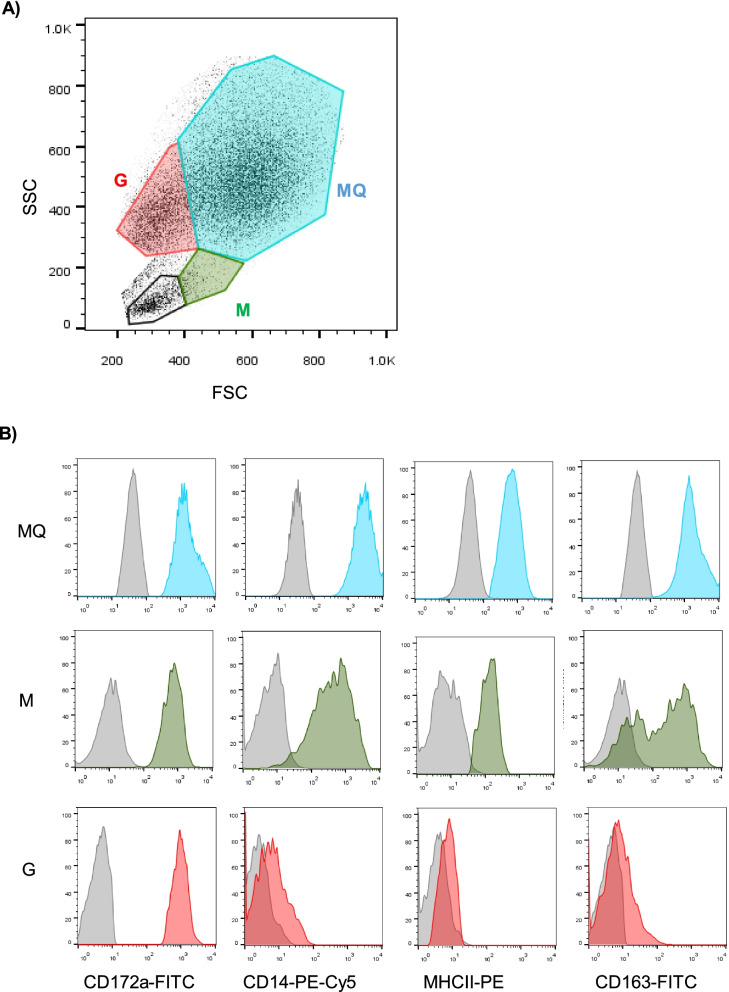
Flow cytometric analysis of CD172a, CD14, CD163, and MHC-II expression on BALF myeloid cells. BALF leukocytes were labeled with monoclonal antibodies to the cell antigens CD172a, CD14, CD163, and MHC-II, and labeled cells were analyzed by flow cytometry. **A** BALF macrophages (MQ), granulocytes (G), and monocytes (M) were identified based on their SSC and FSC properties. **B** The cell-type-specific staining with monoclonal antibodies or isotype controls was shown for all cell types as histograms

The expression pattern of the myeloid cell marker CD172a (Signal regulatory protein α; SIRP α), the LPS receptor CD14, the scavenger receptor CD163, and the antigen-presenting receptor major histocompatibility complex (MHC)-class II molecules was analyzed for BALF macrophages, granulocytes, and monocytes in healthy and diseased camels (Fig. 5A-B). While CD172a was expressed on all cell types, only macrophages and monocytes expressed CD14, CD163, and MHC-II (Fig. 5A-B).

**Fig. 6:**
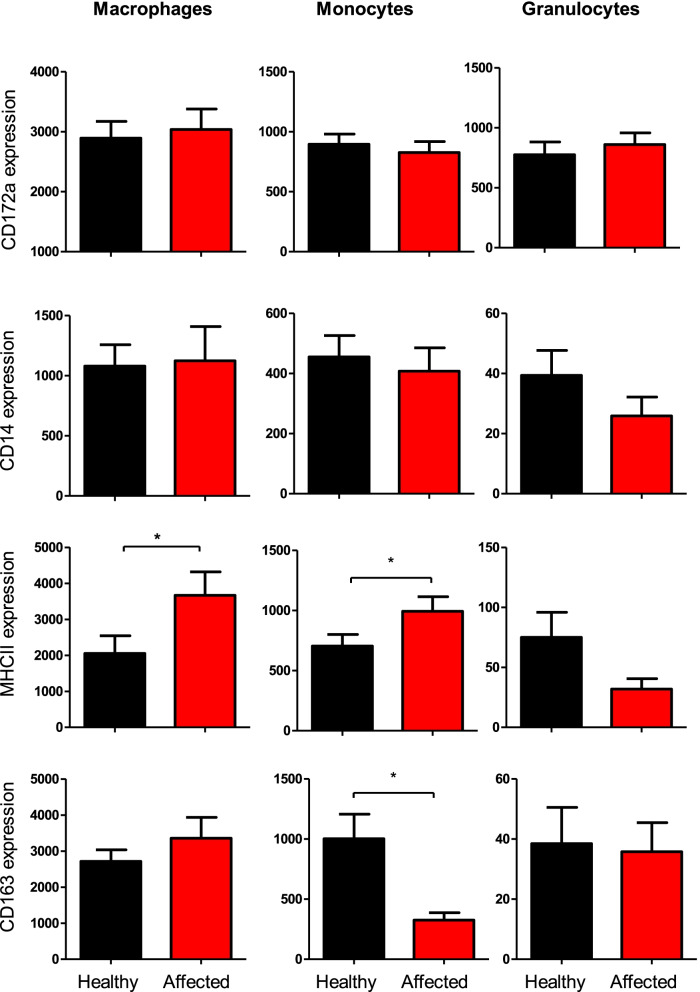
The expression density of the cell markers CD172a, CD14, CD163, and MHC-II on BALF myeloid cells from healthy and diseased camels. BALF leukocytes were labeled with monoclonal antibodies to the cell antigens CD172a, CD14, CD163, and MHC-II, and labeled cells were analyzed by flow cytometry. The expression density of all cell markers was calculated as mean fluorescence intensity (MFI) values and data were presented graphically for macrophages, granulocytes, and monocytes. * = *p* < 0.05

For all cell types, the expression levels of CD172a and CD14 did not differ significantly between healthy and diseased camels. BALF macrophages and monocytes from diseased camels showed higher expression of MHC-II molecules compared to cells from healthy animals. Only for BALF monocytes, CD163 expression was significantly lower in diseased than healthy camels (Fig. 6).

## Discussion

Respiratory diseases are among the most common and expensive to treat diseases in camels with high impact on camel health, welfare, and production. Understanding the immune cell dynamics in the respiratory mucosa is necessary for efficient management of camel respiratory diseases. Bronchoalveolar lavage fluid (BALF) has been proven as a valuable sample for investigating mucosal immune cells in the respiratory tract of several species. In the present study, fluorescent antibody labeling and flow cytometry were used to study the immune cell composition of BALF in dromedary camels. Animals with clinical respiratory diseases were compared with apparently healthy animals. In addition, blood leukocytes from the same animals were also stained with the same antibodies and analyzed by flow cytometry.

Based on their forward (FSC) and side (SSC) light scattering characteristics, which are indicative of cell size and granularity, respectively, the immune cell populations in the camel BALF were classified into FSC^high^ SSC^high^ macrophages, FSC^low^ SSC^high^ granulocytes, FSC^high^ SSC^low^ monocytes, and FSC^low^ SSC^low^ lymphocytes. Similar cell populations were identified in the bovine BALF based on the same light-scattering properties [48, 57]. The dominance of macrophages (~ 70%) with smaller proportions for granulocytes, monocytes, and lymphocytes, indicates the similar composition of the immune cell populations in camel and bovine BALF [48, 57]. Similar results were also previously obtained by microscopic analysis of BALF cytology in camels [29].

In camels with respiratory disease, BALF samples contained higher total immune cell numbers than healthy camels, which is similar to findings in respiratory diseased cattle and horses [15, 22, 60–62]. Similarly, the decrease in macrophages with expansion in the fraction of granulocytes is also in line with findings in respiratory diseased cattle [21], horses [14, 60], donkeys [61], and other camelids [24]. Although the proportion of the total lymphocyte population in camel BALF was not affected by respiratory disease, BALF samples from diseased camels contained higher percentages of CD4+ T cells and B cells, than healthy animals. The higher abundance of the cell adhesion molecule LFA-1 on BALF CD4+ T cells from diseased animals indicates the higher presence of CD4+ T cells that are antigen specific and expanded due to infection. Studies on the cellular composition of BALF in humans and experimental animals reported the presence of alveolar macrophages in the alveolar space under steady-state conditions, while, neutrophils and lymphocytes are recruited to the respiratory tract upon infection or injury. Interferon-gamma (IFN-γ), tumor necrosis factor-alpha (TNF-α), and interleukin (IL)-1β were reported as inflammatory cytokines involved in the lung inflammatory response [35, 63]. In the dromedary camel, the identification of immune mediators including cytokines and chemokines that are involved in the observed change in immune cell composition is not yet investigated.

Staining of camel BALF leukocytes with monoclonal antibodies to the myeloid cell marker CD172a (Signal regulatory protein α; SIRP α) [64], the LPS receptor CD14, the scavenger receptor CD163, and the antigen-presenting receptor major histocompatibility complex (MHC)-class II molecules identified these cell markers as a valuable tool for the immunophenotyping of myeloid cell population in camel BALF. While CD172a was expressed on all cell types, only macrophages and monocytes expressed CD14, CD163, and MHC-II, implying a similar phenotype of myeloid cells in camel, bovine, and porcine BALF [48, 57, 65].

Alveolar macrophages are essential cells that contribute to the innate defense mechanism in the lungs by mediating a pro-inflammatory immune response and elimination of pathogens through phagocytosis. Moreover, these cells can mediate an anti-inflammatory immune response to restore tissue homeostasis [66, 67]. They are characterized by plasticity, being able to change their phenotype and function depending on the inflammatory conditions [66, 68]. In the present study, the higher abundance of MHCII molecules on BALF macrophages and monocytes from diseased camels compared to cells from healthy animals indicates a polarization toward the M1 phenotype in respiratory diseased camels. This is also supported by the decreased expression of the M2 marker CD163 on BALF monocytes from diseased camels.

In contrast to the observed changes in BALF immune cell composition, blood samples from healthy and respiratory affected camels contained similar numbers of total leukocytes with comparable fractions of granulocytes, lymphocytes, and monocytes in the two animal groups. This was also the case for blood lymphocytes with no changes in the fraction of helper T cells, B cells, and γδ T cells between the healthy and diseased camels. In a recent study, experimental infection with *Chlamydia psittaci* (*C. psittaci*) resulted in significant changes in the immune cell composition and phenotype in BALF of bovine calves*.* In addition, the infection-induced changes in the phenotype of blood monocytes, neutrophils, and T cells, characterized by enhanced expression of activation markers and adhesion molecules, contributed to the rapid eradication of the infection [48]. Although the results of the present study argue against the effect of respiratory disease in camels on blood leukocytes, further studies are required to evaluate the role of the systemic cellular immune system in the local immunity on mucosal surfaces of the respiratory tract in camels.

Although the BALF samples from diseased camels were tested negative for two common respiratory viruses (BPV-3 and BRSV), we cannot exclude infection with other respiratory viruses. In addition, the samples were not tested for bacterial pathogens, which is a limitation of the present study. Therefore, further studies are required with detailed pathogen detection and a higher number of animals to evaluate the pathogen-specific changes in the cellular composition of camel BALF.

## Conclusions

The current study represents the first report on using flow cytometry for the analysis of immune cell composition of bronchoalveolar lavage fluid (BALF) in dromedary camels. Camel BALF macrophages, granulocytes, monocytes, and lymphocytes were identified based on their forward and side scatter properties. The expression pattern of the cell markers CD172a, CD14, CD163, and MHCII molecules on BALF cells indicates a similar phenotype for camel, bovine, and porcine BALF myeloid cells. The comparison between camels with respiratory disease and healthy camels regarding cellular composition in their BALF revealed a higher total cell count, a higher fraction of granulocytes, and a lower fraction of macrophages in diseased than healthy camels. Within the lymphocyte population, the percentages of helper T cells and B cells were also higher in diseased than healthy camels. The elevated expression of the activation marker CD11a on helper T cells of diseased camels indicates a higher frequency of effector helper T cells in the inflamed respiratory tract. The higher abundance of MHCII molecules on BALF macrophages from diseased camels indicates a polarization toward an inflammatory macrophages phenotype (M1) in respiratory diseased camels. No significant differences were observed in the systemic leukogram between healthy and diseased animals.

## Data Availability

The datasets generated and/or analyzed during the current study are not publicly available due restrictions by the owners but are available from the corresponding author on reasonable request.

## Abbreviations

PBS: Phosphate-buffered saline
*S. aureus*: *Staphylococcus aureus*
ROS: reactive oxygen species
MHC: Major histocompatibility complex
CD: Cluster of differentiation
MFI: Mean fluorescence intensity
BALF: Bronchoalveolar lavage fluid
LFA-1: Lymphocyte function-associated antigen 1
RBC: Red blood cell
